# Alignment of magnetic sensing and clinical magnetomyography

**DOI:** 10.3389/fnins.2023.1154572

**Published:** 2023-05-18

**Authors:** Negin Ghahremani Arekhloo, Hossein Parvizi, Siming Zuo, Huxi Wang, Kianoush Nazarpour, Justus Marquetand, Hadi Heidari

**Affiliations:** ^1^Microelectronics Lab, James Watt School of Engineering, The University of Glasgow, Glasgow, United Kingdom; ^2^Neuranics Ltd., Glasgow, United Kingdom; ^3^School of Informatics, The University of Edinburgh, Edinburgh, United Kingdom; ^4^Department of Neural Dynamics and Magnetoencephalography, Hertie Institute for Clinical Brain Research, University of Tübingen, Tübingen, Germany; ^5^MEG Centre, University of Tübingen, Tübingen, Germany; ^6^Department of Neurology and Epileptology, Hertie Institute for Clinical Brain Research, University of Tübingen, Tübingen, Germany

**Keywords:** electromyography, magnetomyography, motor unit decomposition, optically pumped magnetometer, tunnel magnetoresistance, spintronic sensors, superconducting quantum interference devices, wearable sensors

## Abstract

Neuromuscular diseases are a prevalent cause of prolonged and severe suffering for patients, and with the global population aging, it is increasingly becoming a pressing concern. To assess muscle activity in NMDs, clinicians and researchers typically use electromyography (EMG), which can be either non-invasive using surface EMG, or invasive through needle EMG. Surface EMG signals have a low spatial resolution, and while the needle EMG provides a higher resolution, it can be painful for the patients, with an additional risk of infection. The pain associated with the needle EMG can pose a risk for certain patient groups, such as children. For example, children with spinal muscular atrophy (type of NMD) require regular monitoring of treatment efficacy through needle EMG; however, due to the pain caused by the procedure, clinicians often rely on a clinical assessment rather than needle EMG. Magnetomyography (MMG), the magnetic counterpart of the EMG, measures muscle activity non-invasively using magnetic signals. With super-resolution capabilities, MMG has the potential to improve spatial resolution and, in the meantime, address the limitations of EMG. This article discusses the challenges in developing magnetic sensors for MMG, including sensor design and technology advancements that allow for more specific recordings, targeting of individual motor units, and reduction of magnetic noise. In addition, we cover the motor unit behavior and activation pattern, an overview of magnetic sensing technologies, and evaluations of wearable, non-invasive magnetic sensors for MMG.

## 1. Introduction

Skeletal muscles are the primary organs for managing force generation and movements. Smooth control of muscle movements relies on the complex interaction between the neuronal system and the skeletal muscle, called the neuromuscular system. A recent analysis of the Global Burden of Disease (GBD) data showed that approximately 1.71 billion people globally live with long-term neuromuscular diseases, leading to dramatic and long-term suffering for them and their carers (Cieza et al., 2020). As such, a better understanding of anatomical architecture and electromechanical properties of the neuromuscular system is central to diagnosing and monitoring neuromuscular disorders, motor rehabilitation, robotics, and prosthetics.

**Figure 1:**
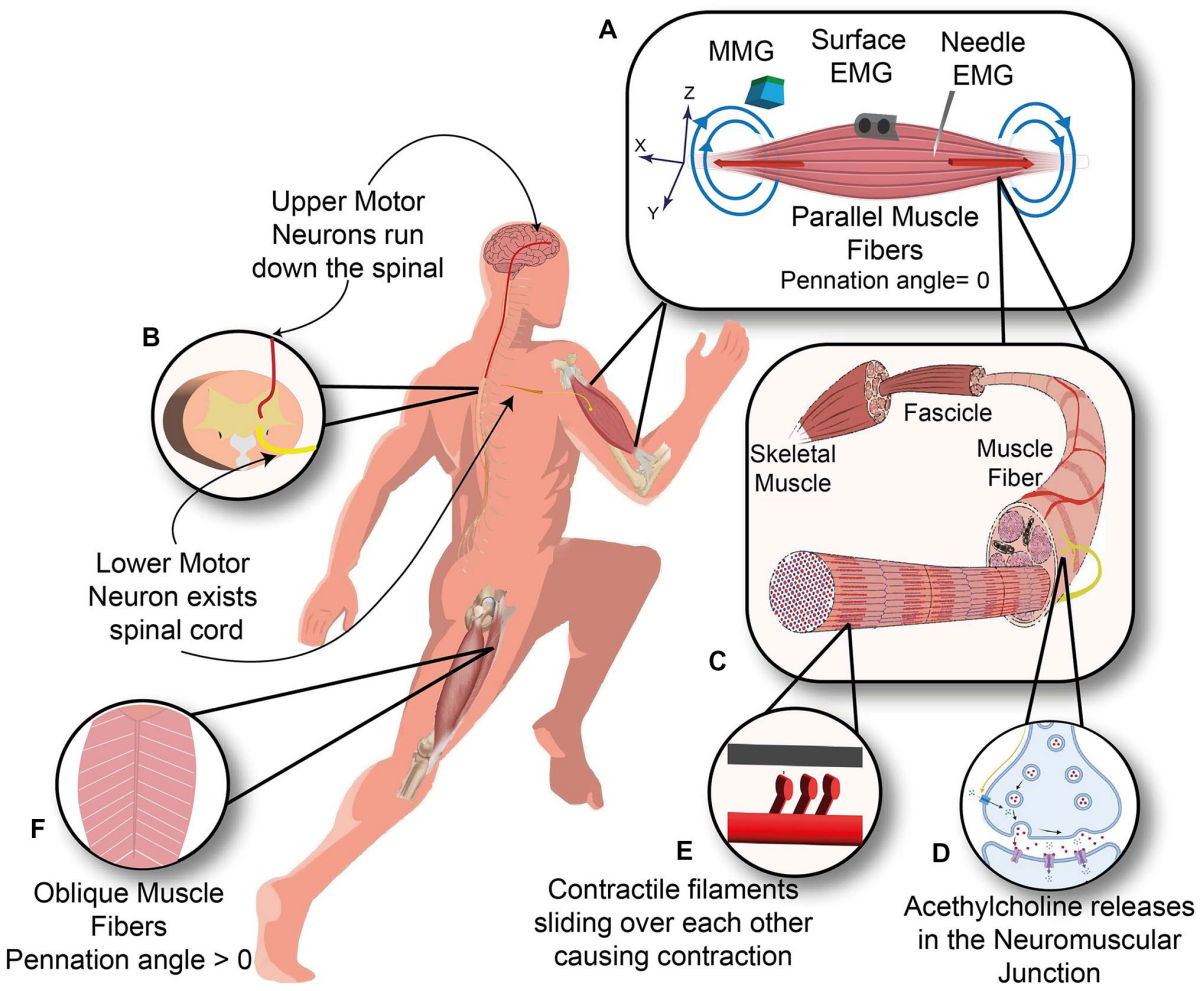
Neuromuscular system. **(A)** Three kinds of sensors measure the Biceps muscle with the parallel orientation of muscle fibers: Needle and surface EMG measuring the electrical current (red arrows) and Magnetomyography sensor measuring the magnetic signals (blue arrows). **(B)** Upper motor neuron descends along the spinal cord and synapses with the lower motor neuron inside the spinal cord. **(C)** Anatomy of skeletal muscle from Fascicle to muscle fibers. **(D)** Neuromuscular junction, where motor neuron meets the muscle fiber. **(E)** Contractile filaments in each muscle fiber slide over each other to cause a contraction. **(F)** Oblique arrangement of muscle fibers in rectus femoris muscle.

When the goal is to extract information from the neuromuscular system, one can interface the neuromuscular system at different levels, e.g., the brain, spinal cord, peripheral nerves, and skeletal muscle, as shown in Figure 1. Among other available options, muscle interfacing is the most viable way to capture the movement intent and neural drive.

Traditionally, muscle activity is recorded electrically using electromyography (EMG), which is the summation of muscle fibers depolarization signals. The first recording of the muscle’s electrical activity was made in 1890 by Marey, who introduced the EMG term (Clarys, 1994), which started with Francesco Redi’s study with the deduction that the electrical shock from Ray fish was muscular in origin in the 1660s (Clarys, 2000). Since then, advances in EMG have significantly impacted healthcare technologies such as rehabilitation and biofeedback, as well as human-machine interfacing (HMI) (De Luca, 1997; Merletti and Farina, 2016).

The EMG signal is a summation of action potentials from active motor units residing in the electrode pick-up area on the skin surface. Moreover, in surface EMG, the recorded signals are separated from the signal source by various biological tissues, most importantly subcutaneous fat, with different electrical conductivities causing dispersion, distortion, and filtering of the propagating signal to the skin surface called the volume conduction effect (Hogrel, 2005). Therefore, the surface EMG signal suffers from a low spatial resolution, less than 2 cm, and it rarely could give any information from the individual motor unit (Kleine et al., 2007).

**Figure 2:**
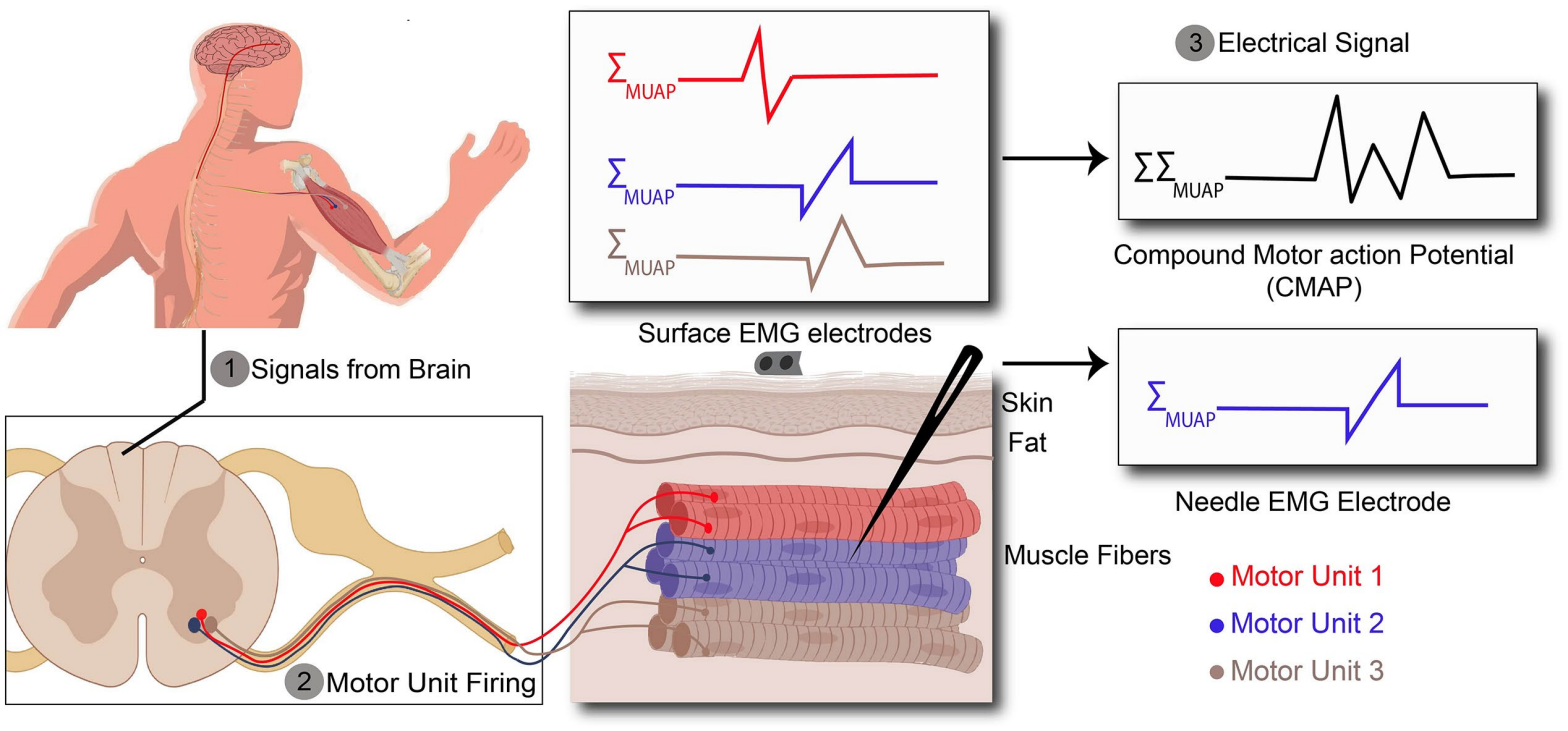
Action potentials (AP) recorded by surface and needle EMG. **(1)** AP are originated in the brain and propagates along the spinal cord. **(2)** AP exiting the spinal cord through the ventral aspect of the spinal cord to the target muscle. **(3)** Recorded signal by surface EMG is a kind of compound signal which is the combination of all active motor unit action potentials (MUAPs), whereas needle EMG signals arise from one motor unit action potential.

To enhance the spatial and temporal resolution, EMG can be recorded using needle EMG electrodes that penetrate the electrically isolating skin and subcutaneous fat and directly measures the muscle action potential with a high spatial resolution, as shown in Figure 2 (Mezzarane et al., 2013). In neuromuscular disorders (NMDs), all muscle fibers and motor units within a muscle are not affected equally, so it is necessary to assess a wide area within the affected muscle by using needle movements through the depth of the muscle which can cause pain and discomfort in the patient. Even though for minimizing patient discomfort and maximizing MUAP collection, appropriate slight needle movement <1 mm is recommended, it can still be performed just in a few locations with few needle insertions, especially in children and patients with lower pain thresholds. Hence, the pain associated with the needle EMG can pose a risk for certain patients. For example, children with spinal muscular atrophy require regular monitoring of treatment efficacy through needle EMG, but due to the pain caused by the procedure, clinicians often rely on a clinical assessment rather than needle EMG.

Besides being a demanding technique due to the needle insertion requirements, few sampling locations, and being painful, needle EMG causes risks. Although it is rarely thought to be associated with severe complications, inserting a needle is invasive, and it has the potential to be associated with iatrogenic complications such as accidental needle sticks, infections, bleeding, and hematoma formation.

Further, the electrical contact, which is essential in both surface and needle EMG to record electrical fields, can cause challenges in clinical applications. First, the quality of electrical contact between the electrode and the skin interferes with the recorded electrical signals. Next, the metal-tissue interface in implanted electrodes can lead to chronic inflammation, fibrosis formation, and finally rejection of the host. Moreover, the electrical currents originating from the metal-tissue interface can also mask direct currents arising from muscles, preventing the diagnosis of several NMDs (Cohen and Givler, 1972).

Surface EMG is a non-invasive method and offers comfortable use. However, its low spatial resolution limits its wide use in clinical settings—e.g., as for NMD diagnosis and post-treatment monitoring, detailed information on the structure and function of a single motor unit is required. In an evidence-based review by Meekins et al. (2008), there was insufficient proof to determine the preference for surface EMG over needle EMG in diagnosing NMDs and distinguishing between neuropathic and myopathic disorders(Meekins et al., 2008). Overall, surface EMG application is limited in clinical practice, and painful needle EMG is the gold standard method for evaluating NMDs; hence alternative non-invasive recording methodologies are necessary. As such, other technologies, like Magnetomyography, Forcemyography, Phonomyography, and Sonomyography, which are based on various signal types (biomechanical, biochemical, and bioelectrical), have been extensively studied in recent years, as shown by Wang et al. (2022).

Magnetomyography (MMG) is one of such non-invasive alternatives for muscle activity recordings, as shown in Figure 1A. MMG records the magnetic field generated by skeletal muscle, which was first proposed by Cohen and Gilver (1972) and defined as the recording of one component of the magnetic field vector versus time, where the magnetic field at the point of measurement is due to currents generated by skeletal muscle (Zuo et al., 2020). The correspondence between EMG and MMG signals originates from the Maxwell-Ampère law, which states that time-changing electrical current densities generate a magnetic field.

MMG signal strength is on the scale of Pico () to Femto () Tesla depending on the measurement circumstances, whether the MMG sensors are implanted in the muscle or applied on the skin outside of the body, respectively. So, compared to the amplitude of the EMG signal, which is on the scale of milli-volts, detecting ultra-low magnetic signals is challenging. However, there are two key drivers in employing magnetic sensors rather than electrical sensors for detecting muscle activities. First, unlike electrical signals, magnetic signals have the advantage that their signal strength is minimally affected by the surrounding tissues like skin and subcutaneous fat, as the body’s tissues are effectively transparent to magnetic fields (Arekhloo et al., 2022). Consequently, magnetic signals can be coupled to the electrical current flowing in the muscle fiber and offer significantly higher spatial resolution (~mm). Secondly, magnetic sensors also do not need contact to record signals. Hence, the problems associated with using electrodes, such as poor electrical contact, interfering direct current (DC), contact potentials arising from both surface and needle EMG, and inability to be used in chronic implants, are non-existent (Sternickel and Braginski, 2006). Therefore, MMG can improve long-term biocompatibility if the corresponding sensors will be packaged with biocompatible materials. Another advantage of MMG over EMG is raised from inherently vector quantities of the magnetic field that could assist in recording muscle movement. As such, while EMG is restricted to the plane of skin and is not yielding any vectorial information, MMG signals are totally sensitive to the signal direction, which can be used as an additional source of information for understanding muscle physiology (Parker and Wikswo, 1997). In this review article, we will discuss the basics of magnetic muscle signals and the various types of successful magnetic sensors that have been developed. We will also examine the engineering specifications of these sensors, as well as the clinical experiments that have been conducted using them. Additionally, we will explore the evolution of these technologies and how they have progressed over time. Overall, this article will provide a comprehensive overview of the field of magnetic muscle signal detection and its current state of development.

## 2. Methodology

In the peripheral neuromuscular system, the motor unit is considered a basic functional unit that is responsible for signal production. Each motor unit consists of a motor neuron, including its dendrites and axons, and the muscle fibers innervated by the motor neuron (Duchateau and Enoka, 2011). Motor units have their own characteristics such as innervation ratio, motor unit action potential (MUAP), firing frequency rate, etc. This information is usually obtained through needle EMG since it can record a single motor unit with high spatial resolution due to the needle placement near the muscle fibers, as illustrated in Figure 2. In contrast to needle EMG, there are also several studies which have shown the possibility of surface EMG (High-density surface EMG) for evaluating single motor unit behavior (Disselhorst-Klug et al., 1998; Merletti et al., 2008; Holobar et al., 2009), but as it requires computational management and complicated signal detection, it is not viable for daily clinical practice. This is due to the fact that-unlike the needle EMG - surface EMG signals are the spatiotemporal summation of the MUAP from all engaged motor units, which leads to the necessity of more post-processing of the data.

### 2.1. Motor unit behavior

To understand the actual signal source/generator of the muscle—the motor unit—better, one has to consider the underlying anatomy and physiology of the neuromuscular system: As shown in Figure 2, when the muscle cells are required to contract, they receive inputs from the large cells lying in the ventral aspect of the spinal cord called motor neurons. A motor neuron with its extensive branches of dendrites located in the ventral root of the spinal cord receives excitatory and inhibitory inputs from other parts of the nervous system. These inputs are integrated, and if the summation of these inputs exceeds a critical voltage threshold, an action potential is generated at the beginning of the axon (so-called axon hillock) in a motor neuron. The generated action potential exits the spinal cord and propagates along the entire length of the axon to trigger muscle fibers yielding in muscle fiber contraction (see also 2.2 motor unit activation). Muscle fibers are a variety of highly organized elongated cells that group together and build up skeletal muscle.

Muscle fibers are electrically excitable as their cell membrane can produce electrical activities in response to external stimuli and conduct them along the muscle membrane leading to muscle contraction via a complex biomechanical cascade (Malmivuo and Plonsey, 1995). So, in each motor unit, motor neuron activation leads to a synchronous contraction of all innervated muscle fibers. Therefore, the summation of the action potential of all muscle fibers belonging to a motor unit is called motor unit action potential (MUAP).

The number of muscle fibers innervated by each motor neuron (known as innervation ratio) varies widely, ranging from a few muscle fibers in extraocular muscles to thousands of muscle fibers in large limb muscles (Enoka, 1995). There is a significant association between the motor neuron size, the innervation ratio of the involved motor unit, and its force output (Clamann, 1993). As such, motor units with larger motor neurons have a higher innervation ratio and could exert a stronger contraction force level (Gordon et al., 2004). As an example, a motor unit of the rectus lateralis muscle (eye muscle) has an innervation ratio of 5 and a motor unit of the medial gastrocnemius muscle (leg muscle) has an innervation ratio of 2000 muscle fibers (Enoka, 1995). Logically, a synchronous contraction of 2000 muscle fibers leads to higher force output compared to the 5 muscle fibers contraction.

**Figure 3:**
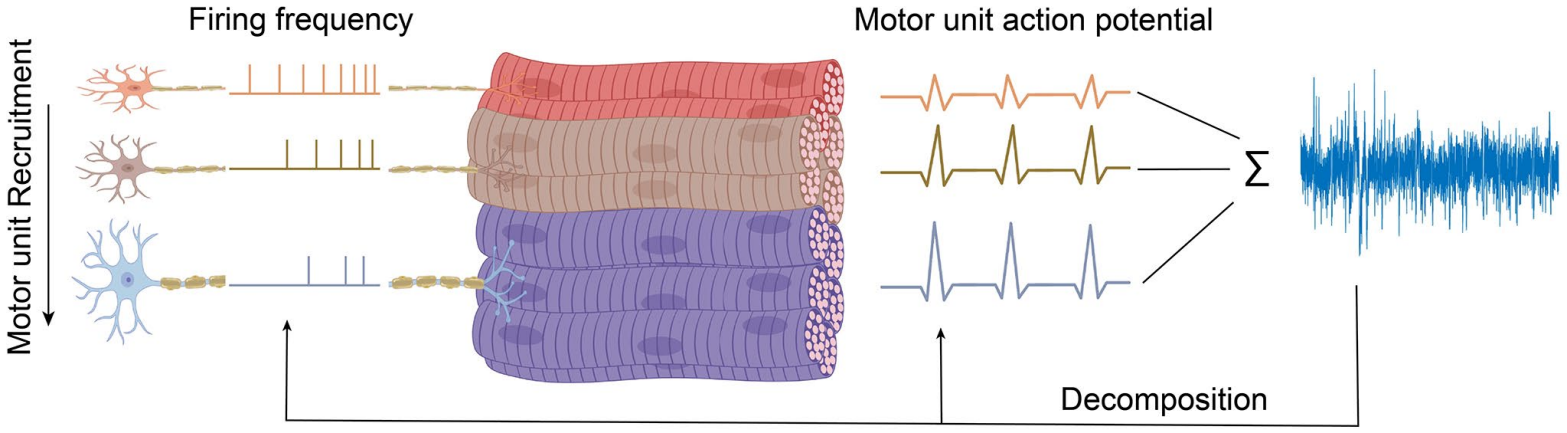
Motor unit decomposition. Decomposing signals to individual motor unit.

For maintaining or generating more muscle force, new motor units following a particular and fixed sequence are coming into play (recruited), which is illustrated in Figure 3. The force level at which a new motor unit will be activated is called the recruitment threshold. According to this, small motor units with lower thresholds are activated earlier, and the larger motor units with higher thresholds enter the contraction later. This principle was first introduced by Henneman and Mendell (2011), which declares that the sequential recruiting of motor units in a contraction cycle is inconsistent with increasing motor unit size and innervation ratio.

The force that a muscle generates does not only rely on which or how many motor units have been recruited but also at what rate they are firing action potentials, called rate coding (Enoka, 1995). The action potential frequency increases with increasing muscle force and decreases with muscle relaxation. During each contraction cycle, motor units that switched on earlier will reach the highest discharge frequency (Person and Kudina, 1972). So, firing rates are inversely proportional to the recruitment threshold of a motor unit, as illustrated in Figure 3. Thus, when muscle force is increased, besides recruiting new motor units, the firing frequencies of all active motor units also increase, as shown in Figure 3. The relative contribution of recruitment and rate coding strategies to muscle strength depends on the type of muscle, muscle task, and the type of contraction. Hence, a comprehensive understanding of the underlying physiological mechanisms responsible for generating or maintaining muscle force is necessary to study the contribution of the individual motor unit to the recorded EMG signals.

The ability to define individual motor unit contribution to the recorded signal (called motor unit decomposition) will shed light on diagnosing and monitoring upper motor neuron disorders like amyotrophic lateral sclerosis (ALS), stroke (De Luca et al., 2006), neuromuscular disorders (Dorfman et al., 1989), exercise-induced fatigue (Merletti et al., 2003), and neuroprosthetics fine control (Merletti et al., 2003).

### 2.2. Motor unit activation

**Figure 4:**
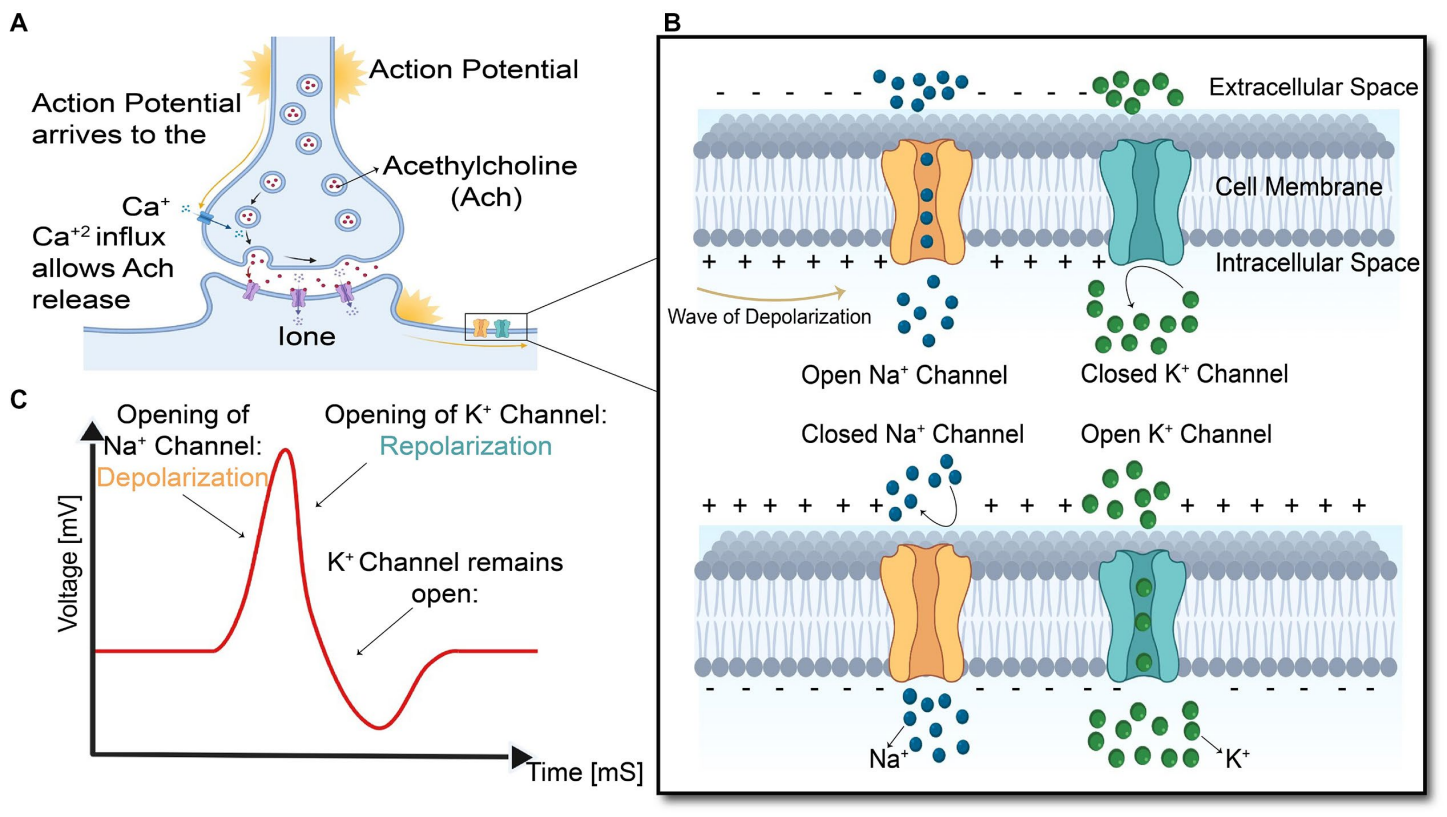
Summary of the events in the generation and propagation of action potential in a skeletal muscle fiber. **(A)** End-plate potential at Neuromuscular junction, **(B)** generation and propagation of action potential, and **(C)** action potential diagram.

As shown in Figure 4, the arrival of action potential to the motor neuron endplate causes the release of the neurotransmitter acetylcholine into the gap between the nerve and muscle fiber. After Acetylcholine diffuses across the gap to the muscle membrane, it binds to its specialized receptors on the muscle cell membrane leading to an inward flux of sodium and calcium, which shifts membrane potential toward the positive values and threshold. If the membrane voltage exceeds its threshold, a high density of voltage-gated sodium channels will be activated, and sodium ions start flushing into the muscle cells and eliciting an action potential which is called depolarization of the muscle membrane. Opening voltage-gated potassium channels after closing voltage-gated sodium channels causes an efflux of potassium from the muscle cell, which reverses the membrane potential to the resting state called repolarization (Fletcher, 2011). These coordinated changes in muscle polarity caused by ion currents through the cell membrane are called muscle fiber action potential (MFAP).

**Figure 5:**
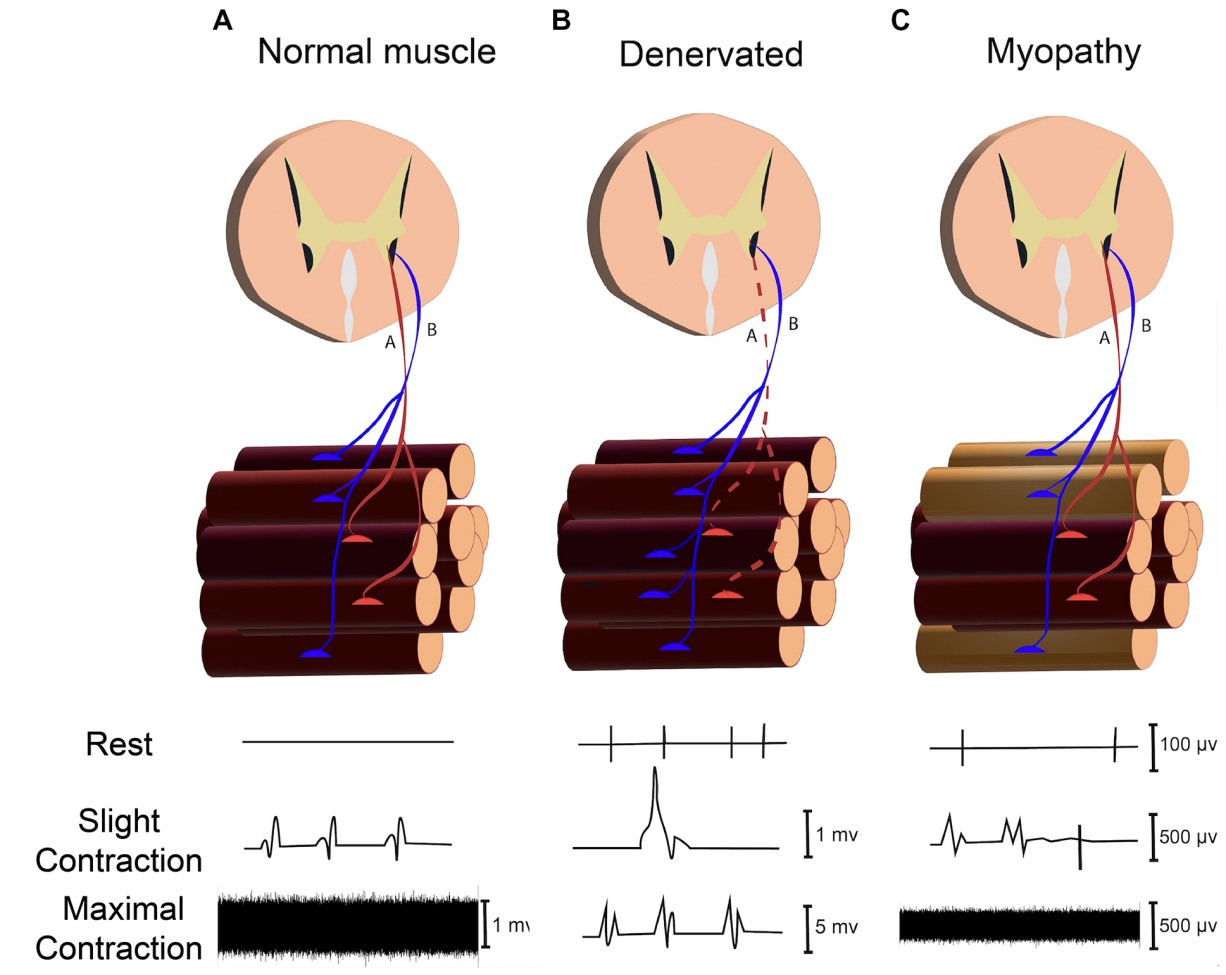
Assessing neuromuscular disorder through recording signals in rest (Spontaneous activity), slight and maximal force contraction (MUAPs) in **(A)** Normal Muscle. **(B)** Denervated Muscle. **(C)** Myopathy.

This muscle fiber action potential in a small section of muscle membrane provokes the same sequential opening of voltage-gated sodium and K channels of the neighboring membrane patch on both the right and left sides. The action potential thus originates from the muscle center called the innervation zone and propagates as a wave in opposite directions toward the two tendon endings of muscle fibers (Farina and Merletti, 2004). While action potential is running across the muscle cell membrane, spreading into the interior of the muscle cell via T tubules (specialized membrane radially oriented invagination into the muscle cell with a high number of ion channels), leading to calcium release from the sarcoplasmic reticulum, which is located closely opposed to T-tubules (Kuo and Ehrlich, 2015). Calcium release from the sarcoplasmic reticulum triggers the interaction of intracellular contractile elements (Actin and Myosin filaments), resulting in muscle contraction, illustrated in Figure 1E and Figure 5.

The transmembrane current caused by inward and outward ions flux during the action potential provokes changes in the external electrical field, which can be measured by employing extracellular microelectrodes. Propagation of action potential causes an intracellular potential gradient which makes ions flow at the front and back of the innervation zone, yields in an axial intracellular current. Compared to the transmembrane current, the intracellular axial current mainly contributes to the net magnetic field produced by muscle activities (Barbieri et al., 2016). So, both electrical and magnetic biosignals originate from the same underlying electrophysiological events.

Any muscle weakness can arise from any alteration in the entire neuromuscular system from activation of the brain cortex, propagation of commands from the brain to the anterior aspect of the spinal cord (anterior horn cell), nerve root, activation of the motor axon, neuromuscular junction, and muscle fibers themselves, as shown in Figure 1. EMG and MMG findings can provide information about the innervating motor neurons, neuromuscular junction, and muscle fibers in a motor unit.

**Figure 6:**
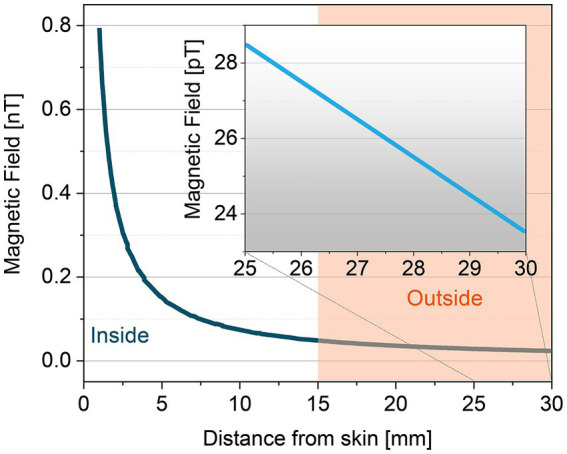
Distance dependence of MMG signals from inside and outside of the skin.

The magnitude of the MMG signal declines with the third power of the distance between the transducer and the current source. As such, the magnetic signal magnitude is: (1) 0.5 to 1.5 nano-Tesla, when the sensor is attached to the skin (2) 20 to 500 pico-Tesla, when the sensor is 10–15 mm away from the skin, as demonstrated in Figure 6. Hence, one of the challenges in MMG recording is dimensional changes of the target skeletal muscle during contraction or movement as it could affect the distance between sensor and skin surface which changes the magnitude of MMG signals. Consequently, all the human studies *in vivo* collected the MMG signal while volunteers performed isometric contractions.

The whole muscle can be assumed as a group of current-carrying line conductors along with the sensor-sensitive axis. During the actual measurement, the Biot-Savart law can be utilized to perform minimum analysis calculations on these linear conductors to estimate the magnetic field strength, where the magnetic flux density B(r) at the location $r^{\prime }$ can be expressed as below:

(1) $$B\left(r\right)=\frac{\mu_{0}\mu_{r}I_{con}}{4\pi }\int \frac{ds^{\prime }\times \left(r-r^{\prime }\right)}{∥r-r^{\prime }{∥}^{3}}$$

It is noted that the muscle fiber is locally regarded as a current-carrying conductor within a constant current, *I*_con_, and an infinitely tiny length ds’. The signal magnitude principally depends on the distance between a measurement point and the source, explained as $ds^{\prime }.\;r=r\cdot e_{R}$

This cylindrical coordinate is suitable for further calculations with an assumption of a symmetrical conductor. With a linear conductor of length l is along the z-axis with $r^{\prime }=z\cdot e_{z}$, an infinitely short conductor cross-section, thus, can be defined as $ds^{\prime }=dz\cdot e_{z}$. Finally, the total magnetic flux density B(r) can be obtained:

(2) $$\begin{array}{l} B\left(r\right)=B\left(r\cdot e_{R}\right)=\frac{\mu_{0}\mu_{r}I_{con}}{4\pi }\int_{z=0}^{l}\frac{dz\cdot e_{z}\times \left(r\cdot e_{R}-z\cdot e_{z}\right)}{∥r\cdot e_{R}-z\cdot e_{z}{∥}^{3}}\\ \;\;\;\;\;\;\;\;\;\;\;\;\;\;=\frac{\mu_{0}\mu_{r}I_{con}}{4\pi }\int_{z=0}^{l}\frac{r\cdot dz\cdot e_{\varphi }-z\cdot dz\left(e_{z}\times e_{z}\right)}{{\sqrt{r^{2}+z^{2}}}^{3}}\\ \;\;\;\;\;\;\;\;\;\;\;\;\;\;=\frac{\mu_{0}\mu_{r}I_{con}}{4\pi }\int_{z=0}^{l}\frac{r\cdot dz\cdot e_{\varphi }}{{\sqrt{r^{2}+z^{2}}}^{3}}\\ \;\;\;\;\;\;\;\;\;\;\;\;\;\;\;=\frac{\mu_{0}\mu_{r}I_{con}r}{4\pi }{\left[\frac{z}{r^{2}\sqrt{r^{2}+z^{2}}}\right]}_{z=0}^{l}e_{\varphi }\\ \;\;\;\;\;\;\;\;\;\;\;\;\;\;\;=\frac{\mu_{0}\mu_{r}}{4\pi r}\cdot I_{con}\cdot \frac{l}{\sqrt{l^{2}+r^{2}}}e_{\varphi } \end{array}$$

## 3. Engineering of magnetic sensors

With the rapid development of micro-and nanoscale magnetic sensors, non-invasive recording of muscle activity through magnetic measurement has become a promising approach for biomedical applications. Magnetomyographic sensors can provide a promising future for improving medical diagnosis, health monitoring, rehabilitation, robotics, and extended reality in which the human-machine interface (HMI) can assist patients with limb length discrepancy to perform essential activities in daily living (Zuo et al., 2020).

**Figure 7:**
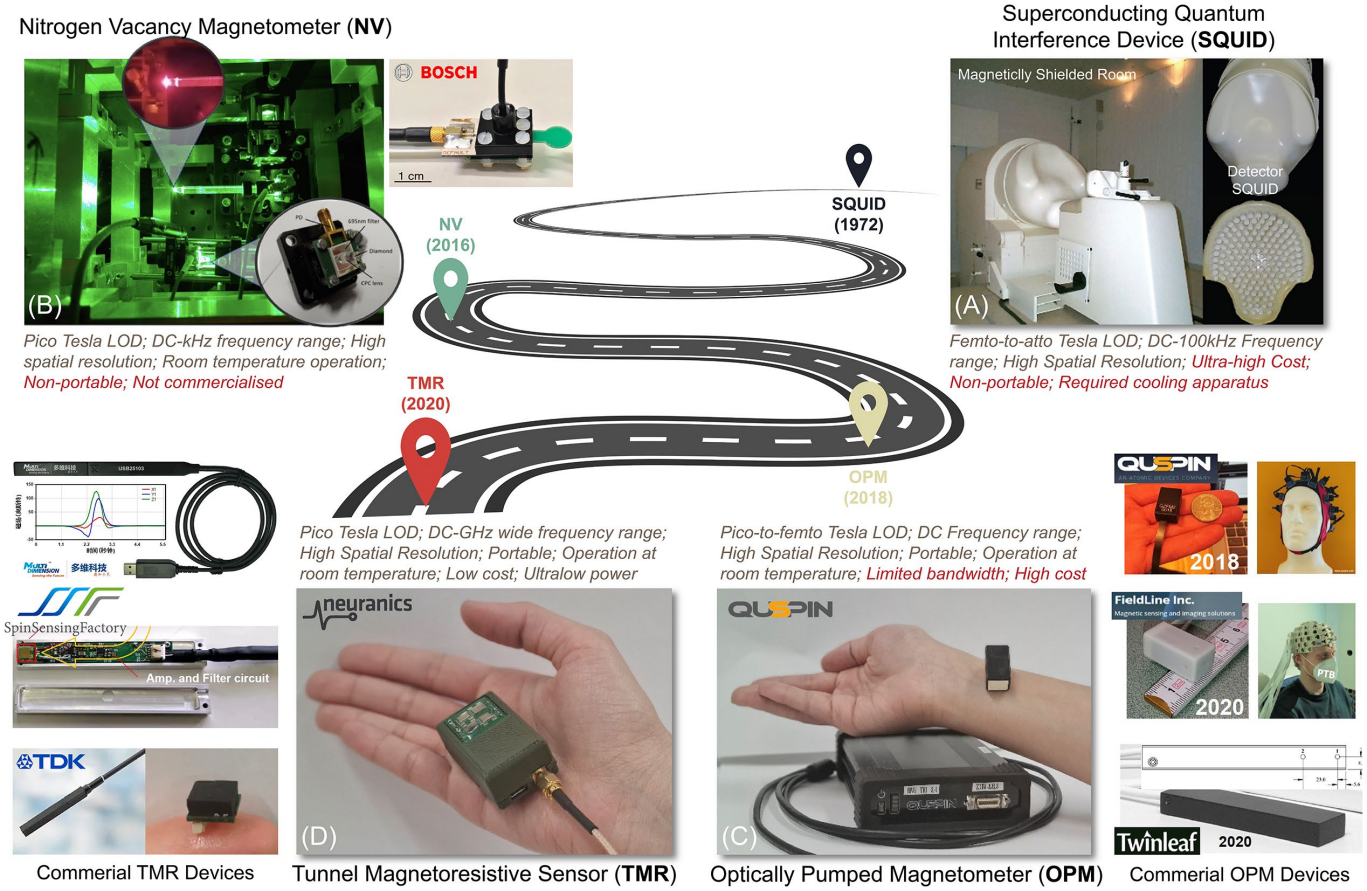
A roadmap for magnetic sensing technologies. **(A)** SQUID, **(B)** NV-based sensor (Kübler, 2023), **(C)** OPM, and **(D)** TMR sensors.

Figure 7 illustrates a progress pathway of MMG sensors from Superconducting Quantum Interference Devices (SQUID), Optically Pumped Magnetometer (OPM), Nitrogen-Vacancy (NV) based magnetometer (Kübler, 2023), to the more recent sensors, Tunnel Magneto Resistance (TMR) sensors.

### 3.1. Superconducting quantum interference devices

The development of SQUID caused a powerful impetus for the improvement of other magnetomyogram sensors, and so far, it is the most sensitive device with Femto-Tesla detection ability and the possibility to achieve atto-Tesla sensitivity with averaging. SQUID combines the physical phenomena of flux quantization and the Josephson effect (two properties of superconductors) to detect small variations in magnetic flux. Magnetic flux in a closed superconducting loop will be quantized in units of the flux quantum (Körber et al., 2016). Josephson’s effect involves two weakly coupled superconductors separated by a tunnel barrier (Kleiner et al., 2004). Currents below a critical value flow between superconductors as a supercurrent, and no voltage is developed across the junction; however, a voltage can be developed for currents above the critical values. Regarding this, dc SQUID includes two Josephson junctions in the parallel configuration on a superconducting loop and operates in the voltage state with a current bias (Jaklevic et al., 1964). When the flux in the loop is raised, the voltage oscillates with a period Φ_0_. By detecting a slight change in the voltage, one can detect a change in flux, typically as low as10^−6^ Φ_0_ (Körber et al., 2016). Almost all commercial SQUIDs are dc SQUIDS because they offer lower noise and higher sensitivity (Kleiner et al., 2004). Many SQUIDs are made of the low-transition-temperature (low Tc), bulky superconductor (Tc) Niobium cooled to liquid-helium temperature, 4.2 K. The advent of high-transmission-temperature (high Tc) thin-film superconductors cooled to liquid-nitrogen is a promising means to reduce sensor-to-muscle separation and intrinsic noise level to improve spatial resolution and high-frequency signal detection (Koelle et al., 1999).

Despite clear evidence of the potential use of SQUID in muscle signal recordings, their high cost, cumbersome weight, lack of spatial flexibility, and large cryogenic apparatus needed for its operation limit the wide use of this technique. In addition, liquid helium or nitrogen required for setting up SQUID sensors prevent any possibility of conducting experiments in contact with living tissues yielding poor spatial resolution. This disadvantage also hinders magnetic measurement at the local scale or dissected tissue microscopy.

### 3.2. Optically pumped magnetometer

Optically Pumped Magnetometer (OPM) is a quantum magnetic sensing technology based on the Zeeman effect, which is a shift of energy level in atoms when exposed to a magnetic field (Cohen-Tannoudji et al., 2019). OPM sensors contain glass cells filled with spin-polarized alkali atoms, mainly Rubidium or Helium atoms, laser optics to pump polarized laser light to the glass cells, and a photodiode for detecting the light passing the vapor (Fu et al., 2020). To achieve the necessary vapor form of the alkali atoms, the glass cells must be heated to a specific temperature, such as 150°C in the case of Rubidium atoms. The pump laser light polarizes the alkali vapors, which interact with the external magnetic field and modulate the light passing through the vapor. The strength of the magnetic field can be determined by measuring the light at the photodiode (Brookes et al., 2022). Even though it has been more than 60 years since it was proven that OPM could be used for detecting small magnetic fields (Budker and Romalis, 2007), it has been only used for MMG since 2018. Recently, commercial OPM sensors have become available, including those from competing manufacturers like QuSpin Inc., FieldLine Inc., and Twinleaf (Savukov et al., 2017). Due to their compact size, they can operate in a magnetically shielded chamber instead of a magnetically shielded room. As such, commercial OPMs offer the additional advantage of flexible sensor placement, allowing for closer proximity to the target muscle and thus, higher spatial resolution. Moreover, they do not require cryogenic cooling, are easy to use, and can record signals with a table-top shield instead of magnetically shielded room. However, they may not match SQUID in dynamic range, sensitivity, and frequency bandwidth.

### 3.3. Nitrogen-vacancy magnetometer

Nitrogen-vacancy (NV) based magnetic sensor is a type of quantum sensor that recently has received growing attention for recording magnetic fields. The nitrogen-vacancy center is a defect in a diamond with an electronic structure that has an energy shift induced by an external magnetic field (Wolf et al., 2015; Chatzidrosos et al., 2017; Fescenko et al., 2020). These energy shifts will excite sharp resonances in the intensity of the photoluminescence so that they can be measured optically. NV-based magnetic sensors have a high dynamic range allowing the background field to be recorded without saturation. Since the sensor is not saturated, the records of the background field signals can be removed by adaptive notch filtering. Recent studies have shown that the sensitivity of the NV-based magnetic sensor has achieved 50 $pT/\sqrt{Hz}\;$ and can be applied to detect the magnetic field of the muscle contraction in an unshielded, ambient environment (Webb et al., 2021). One of the key advantages of NV sensors is the biocompatibility of diamonds which helps us to bring NV in contact or within the electrically excitable biological tissue like muscle and neurons. Besides, operating at a distance smaller than the action potential wavelength could enhance the signal-to-noise ratio since the extracellular return current’s contribution decreases significantly with decreasing sensor-to-source separation (Barry et al., 2016).

NV sensors at this stage cannot yet compete with probe electrophysiology in terms of sensitivity and the high-power consumption requirement of the laser. The integration of the whole system also needs to be improved. Nevertheless, these measurements are an early but important step toward MMG signal detection using quantum sensors.

### 3.4. Spintronic sensors

Magnetic sensors based on the spintronics effect have been extensively studied as a promising pico-Tesla biosensing approach at room temperature. Spintronics or spin electronics refers to the study of the integral features of the electrons known as spin. Spintronic materials exhibit spin-related magnetoresistive (MR) effect and have variable electrical resistance. Their electrical resistance depends on the direction and magnitude of the applied magnetic field. The spin-related MR phenomenon led to the advancement of magnetic sensors that have the potential to detect magnetic fields in the pico-Tesla range at room temperature, suitable for MMG recording. The average size of spintronic sensors is substantially lower than that of SQUID or OPM. Magnetoresistive magnetometers are appealing because of their low-cost applications and being simply powered by a constant current. The value for magnetoresistive sensors is usually determined as the percentage change in the resistance per Oe or the voltage change output per Oe. Three main magnetic sensors based on the thin-film MR effect are Anisotropic Magnetoresistance (AMR), Giant Magnetoresistance (GMR), and Tunnel Magnetoresistance (TMR). Under the same external magnetic field, the output of TMR sensors is 20 times higher than that of GMR sensors which is, in-turn, four times larger than AMR sensors. The key characteristic of spintronic sensors is that they can be miniaturized and implemented on the macroscale, which is practical for lab-on-chip applications associated with biomedicine (Table 1).

**Table 1:** A comparison between types of magnetic sensors.

Sensor	Quantum effect	Array config.	Portability	Frequency range	Limit of detection	Size	Cost
SQUID (Körber et al., 2016)	Superconduction effect	Yes	No	DC-100 kHz	Femto-atto Tesla	m^3^	Ultra-high
OPM (Gutteling et al., 2023)	Zeeman effect	Yes	Yes	2 kHz	Pico-femto Tesla	cm^3^	High
NV (Webb et al., 2021)	Photoluminescence	No	No	DC-kHz	Pico Tesla	×10 cm^3^	Not commercialized
TMR (Zuo et al., 2020)	Spintronics	Yes	Yes	DC-GHz	Pico Tesla	mm^3^	Low

#### 3.4.1. Anisotropic magnetoresistance

It was initially observed that the resistance of some materials, like Nickel and Iron, changes according to the angle between the direction of current flow and the magnetization (McGuire and Potter, 1975). As such, in the field, the magnetization rotates toward the direction of the magnetic field. Magnetic sensors based on this phenomenon are called anisotropic magnetoresistance or AMR sensors. Typical AMR values are ≈5% for NiFe and CoFe bulk alloys and ≈2% for patterned thin films at room temperature (McGuire and Potter, 1975) due to additional scattering caused by grain boundaries and other film interfaces. The low magnitude of the AMR effect, its intrinsic bulk properties to sense only the magnitude and not the direction, and nonlinear output are major drawbacks of AMR which encourage the development of GMR and TMR sensors (Zuo et al., 2020).

#### 3.4.2. Giant magnetoresistance

Attempts for developing spin-based sensors began with the invention of the Giant Magnetoresistance (GMR) effect in 1988 (Barbieri et al., 2016). GMR-based sensors consist of thin-film structures composed of alternate ferromagnetic and non-magnetic layers whose electrical resistance depends on the external magnetic field (Barbieri et al., 2016). The sensitivity of GMR sensors is in the nano-Tesla range; thus, averaging was required to enhance the signal-to-noise ratio (SNR). Furthermore, since the sensitivity of GMR-based sensors is not dependent on their size, they are likely to be miniaturized to micro-scales without sensitivity loss which can be adapted for biological preparations (Fermon et al., 2006). So GMR-based sensors are the preferred choice for magnetic signal detection in the sub-nano-Tesla range at room temperature with high spatial resolution.

#### 3.4.3. Tunnel magnetoresistance

Tunneling-Magnetoresistance (TMR) is a phenomenon observed on ferromagnetic spin tunneling junctions (MTJ) consisting of two ferromagnets separated by a thin insulator. One of the ferromagnetic layers is a free layer in which the spin of its electrons is free to change, and the other layer is the pinned or reference layer because its spin orientation is fixed when the device is made. The barrier between the pinned and free layers is thin enough that electrons can tunnel through. By flowing the current from one magnetic layer to the other across this insulating dielectric material, the probability of electrons which are now quantum mechanically tunneling across essentially an insulator depends on the orientation of these magnetic films. In the application of Magnetomyography, TMR is preferred due to its specifications, including operational linear range, sensitivity, power consumption, and size, which surpass those of AMR or GMR. The TMR sensors with the MgO barrier can be considered the most competitive sensors that could improve the level of detection to the pico-Tesla range in room temperature and low-frequency domain. Further development of this technique is highly dependent on the development of isolating the weak biomagnetic signals from background noise and canceling the geomagnetic field in real-time. In addition, to avoid the effects of movements as much as possible, implantable MMG sensors would be more appropriate for human-machine interfacings, such as control of prosthetic limbs, to reduce the effect of muscle movement.

## 4. Clinical applications of magnetic sensors

### 4.1. SQUID magnetometer

#### 4.1.1. Intracellular conductivity and membrane capacitance

For the first time, Van Egeraat et al. (1990) recorded a single muscle fiber magnetic field of the frog gastrocnemius muscle (leg muscle) by toroidal pickup coil. They provided two detailed cellular properties, such as intracellular conductivity and membrane capacitance which can be used to determine the transmembrane action potential without requiring invasive measurement. These two parameters are also important in distinguishing between different motor units and determining the muscle fiber composition of each motor unit (Van Egeraat et al., 1990).

#### 4.1.2. Detecting direct current with MMG

There are several possible origins for direct currents in muscle fibers, such as muscle afterpotentials, ferromagnetic materials under the skin or inside the muscle, persistent muscle currents after repeated contraction, and muscle injury (Clayton and Emery, 2009). So, being able to measure direct current and distinguishing its source can provide more information on muscle physiology. While direct currents from a muscle are usually masked by dc arising from contact potentials of both needle and surface electrodes of EMG, Mackert et al. (1999) demonstrated an injury-related near-dc magnetic field in human muscle and nerve specimens by dc SQUID from a few centimeters, non-invasively and without any electrical contact (Mackert et al., 1999).

#### 4.1.3. Current source intensity in MMG

MMG shows great potential in estimating current intensity independent of the conductivity of the surrounding tissue, which is not possible using electrical recording unless the electrical conductivity of surrounding tissue and distances to the electrodes are known (Wikswo et al., 1980). The electrical current in muscle fiber, which originated from the motor endplate, near the center of the muscle fiber, propagates toward tendons at both muscle fiber endings. Propagation of muscle fiber action potential is associated with magnetic field variation, which allows the quantification of current source intensity and the number of activated muscle fibers within the motor unit by dividing the dipole moment of the current source by the typical dipole moment in a muscle fiber (Masuda et al., 1999).

#### 4.1.4. Squid in pregnancy

The contractile activity of the uterine muscle results from the generation and propagation of action potentials inside the uterus. These action potentials occur in a group referred to as a burst and have been shown to associate with each uterine contraction (Eswaran et al., 2004). The properties of this burst, such as the frequency and duration of the action potential and the number of simultaneous active myometrial cells, are directly linked to the characteristics of the contraction, including its frequency, amplitude, and duration. Electromyography (EMG) techniques have been employed to record the electrical activity of the uterus, both internally and through surface electrodes on the abdominal surface (Wolfs and van Leeuwen, 1979; Devedeux et al., 1993; Buhimschi et al., 1997). Research has shown that uterine EMG activity recorded from the abdominal surface reflects the electrical activity occurring within the myometrium (Buhimschi et al., 1998). However, the transabdominal EMG methodology has limitations due to the fact that the electrical signals reaching the abdominal surface experience a certain degree of attenuation. This is mainly due to the high dependency on tissue conductivity, which ultimately hinders performing simple analysis of the recorded signals. Moreover, there is high intrasubject variability in the signal strength and noise depending on the variance in the conductivity at the skin-electrode interface. Hence, to obtain the maximum signal strength, extra steps must be considered to prepare the electrode sites and minimize skin impedance. However, these limitations can be overcome by using MMG, as MMG recordings are much less dependent on tissue conductivity. Furthermore, there is no need to prepare the measuring site to reduce skin impedance, as MMG signals are detectable outside the skin boundaries without any contact with the body surface. So, pregnant women can simply lean forward with their abdomen overlying the array of a magnetic sensor. SQUID array for Reproductive Assessment, known as (SARA), is a biomagnetic system designed specifically to record transabdominal MMG to study different aspects of maternal-fetus health (Lowery et al., 2003). SARA comprises 151 magnetic sensors strategically positioned to cover the entire maternal abdomen, allowing for recording uterine contractile activity with high spatial–temporal resolution.

#### 4.1.5. Tracking synchronization changes in uterine contractions

There are unsynchronized and infrequent low-intensity uterine activities during the pregnancy that proceed to rhythmic contraction with better synchronization throughout the uterus resulting in the delivery of the fetus. Therefore, determining the uterine synchronization index can be an indicator for quantifying uterine contraction and evaluating the labor process. As such, recording uterine contractile activity with a high spatial–temporal resolution by a 151-sensors SQUID array results in establishing the time delay in propagation of the electrical activity all over the uterus leading to synchronization evaluation (Eswaran et al., 2002). A study (Ramon et al., 2005) investigating synchronization between channel pairs found that the spatial distribution of the synchronization indices changes and follows the periodic pattern of the uterine contraction cycle. Another study (Eswaran et al., 2009) demonstrated an increase in phase synchronization as the mother approaches to labor. Although only a small percentage of pregnant women demonstrate this characteristic, which may be attributed to the insensitivity of the phases in detecting synchrony. Therefore, another research study used synchrony in magnitude rather than phase synchrony, which shows increased peak-amplitude values of magnetic activity correlated to labor development (Govindan et al., 2015).

#### 4.1.6. Prediction of labor in term and preterm pregnancy

During pregnancy, the uterus remains quiescent and suppresses its contractile activity to maintain an environment for the growth and development of the fetus. Several studies have demonstrated that the myometrial activity in the uterus is minimal during most of the pregnancy, and at term, it rises considerably to forcefully expel the fetus (Zahn, 1984; Marque and Duchene, 1989; Devedeux et al., 1993). However, in about 11% of pregnancies, uterine changes occur earlier and yield in preterm labor (Vogel et al., 2018). Preterm labor, known as births before 37 completed weeks of gestation, is the most common pregnancy complication. According to the level and trend in child mortality report in 2017 (Hug et al., 2017), preterm birth complications were responsible for the majority of deaths among children under 5 years old worldwide. These complications accounted for around 16% of all deaths in this age group and 35% among newborns.

Early diagnosis is the key to managing and preventing preterm labor as if the uterus reaches a specific state, the labor is imminent. So, if preterm labor is diagnosed early, medical experts can take steps to halt the labor process or utilize various interventions to enhance the prognosis for premature infants when preventing preterm delivery is unsuccessful. Unfortunately, there is no gold standard method to predict the onset of either term or preterm labor or adequate techniques to monitor the early changes in the uterus that lead to labor (Garfield et al., 1998).

In a study (Eswaran et al., 2004), the SARA system was used to measure the peak amplitude of MMG signals. The results indicated that an increase in the peak amplitude values of the uterine muscle was correlated with the onset of labor within 48 h. All patients except one with an amplitude higher than 8 pT were delivered within 48 h of the last recordings. In contrast, 5 out of 7 patients with an amplitude lower than 8 pT failed to deliver within the same timeframe. These findings suggest that there is an increase in the electromagnetic activity of the uterus as labor progresses.

In another study (Furdea et al., 2009), authors applied Hilbert-wavelet transforms to the recorded abdominal MMG and deduced that as the pregnant woman approaches to labor, the number of bursts per minute and spread of bursts across sensors will increase.

#### 4.1.7. Ability to characterize levator ani muscle (LAM) function

Levator anal muscles (LAMs) are the integral parts of the pelvic floor and are responsible for the structural supporting of pelvic organs, maintaining continence of the bladder and bowel, and supporting of the vagina and uterus (Heilbrun et al., 2010). In a vaginal birth, LAMs are undergoing more stretch than what the skeletal muscles can normally adapt to without significant injury (Patel et al., 2006). This injury yields pelvic floor disorders, including pelvic organ prolapse and fecal and urinary incontinence, which affects up to 25% of adult women (Leijonhufvud et al., 2011). 151-sensor SQUID array was used to assess LAMs contraction (Kegel exercise) during pregnancy and postpartum in primiparous women (The woman that is pregnant currently and with no prior deliveries), which demonstrated lower root mean square (RMS) in postpartum than pregnancy (Escalona-Vargas et al., 2019). This result exhibits the same characteristics as EMG. In the voluntary contraction of LAMs (Kegel exercise), usually, other accessory muscles in the abdomen, perineum, and thigh are interacting, and there is a study that demonstrated the ability of MMG to distinguish isolated Kegel versus interacting Kegel using bandwidth frequency distribution (Escalona-Vargas et al., 2019).

### 4.2. OPM magnetometer

#### 4.2.1. Determining the innervation pattern of hand

There is a variation in the innervation pattern of the intrinsic hand muscles. Determining this variation is important in the diagnosis and treatment of several clinical situations like entrapment syndrome (Demircay et al., 2011) and spinal cord lesions (Chu et al., 2000). In a study (Broser et al., 2018), magnetic fields of intrinsic hand muscles were recorded by OPM sensors after stimulating the ulnar and median nerve at the elbow level. The muscles which are innervated by the median nerve are clearly distinguished from the muscles that are mainly innervated by the ulnar nerve. The problem is that OPM sensors are not small enough to fit the complex anatomical area like the hand with over 20 muscles which can be managed by using small sensors array (Zuo et al., 2020).

#### 4.2.2. Pennation angle and magnetic field vector

Muscle fibers in a skeletal muscle may lie parallel or with an angle relative to the longitudinal axis of muscle, which is called the pennation angle. For example, as shown in Figures 1A,F, in biceps brachii, muscle fibers are parallel to the longitudinal axis (pennation angle = 0), and in the rectus femoris, muscle fibers are oriented oblique (pennation angle>0). In an experiment conducted by Broser et al. (2021), the possibility of determining muscle fibers geometry in the rectus femoris was shown through recording elicited magnetic field of the rectus femoris by hammer reflex to the patellar tendon at knee level. The direction of the recorded magnetic field from each OPM is in good correspondence with the orientation of muscle fibers inside the rectus femoris.

#### 4.2.3. Recording fasciculation

Neuromuscular disorders (NMDs) are a diverse group of disorders that involve varied components of the neuromuscular system, such as the anterior horn cell, peripheral motor neuron, neuromuscular junction, and muscle fibers (Turakhia et al., 2013). Evaluating NMDs by needle EMG comprises recording and analyzing signals in both resting state (spontaneous activity assessment) and voluntary contraction (motor unit action potential evaluation), as illustrated in Figure 5 (Rubin, 2019). In the resting state, muscle fibers (except the end-plate zone) are electrically silent; hence, the presence of electrical signals in a resting condition (known as spontaneous activities) can represent peripheral neuromuscular disorders. Since spontaneous activities (SA) can be generated from dysfunction in different points of the peripheral neuromuscular system, determining the source of SAs is critical for diagnosing and monitoring NMDs. The origin of the SAs can be identified by their distinctive properties, such as amplitude, number of phases, initial deflection, and frequency (Mills, 2005).

For instance, if the source of a spontaneous discharge is a motor neuron, the amplitude of recorded signals is higher than the ones with muscle fiber origin because a motor neuron innervates many muscle fibers. Therefore, the spatial and temporal summation of the signals from all these muscle fibers makes the amplitude of the recorded signal much higher. Spontaneous activities are mainly detectable with needle EMG as skin and subcutaneous tissues act as low-pass filter and prevent the recording of SAs through surface EMG. However, recording Fasciculation, Myokymia, Tetani, Cramp, and Neuromyotonia, which originated from a motor neuron, are more probable through surface EMG or OPM as they provide higher signal-to-noise (SNR) ratio.

Marquetand et al. (2021), tried to record five types of SAs with needle EMG, surface EMG, and OPM. Among five recorded SAs (PSW, fibrillation, complex repetitive discharge, and myotonic discharge), only fasciculation was detected by OPM and surface EMG. Other forms of spontaneous waveforms are currently not detectable as the required bandwidth for recording SA is 10KHz, but the bandwidth of employed OPM is 135 Hz. The other possible reason is the low signal-to-noise ratio of the recorded signals compared to the experimental setup. Among recorded signals, fasciculation is the only SA which is originated from the motor unit yielding stronger signals with higher amplitude, as previously explained, and consequently higher SNR. By contrast, other recorded spontaneous discharges have lower spatiotemporal summation and higher discharge frequency.

#### 4.2.4. Fatigue characteristics in OPM

Muscle fatigue is the inability to produce **maximal force** in response to sustained or repetitive contractile activity. Muscle fatigue is a common nonspecific experience in daily activities, sports, and a wide range of disease states, including neuromuscular disorders (Lou et al., 2010), cardiovascular disorders (Casillas et al., 2006), and malnutrition (Tardy et al., 2020). Its manifestation in several disorders is a key driver to deeply exploring the underlying mechanism, which seems to be manifold. Regarding the origin of fatigue, it can be divided into two categories central and peripheral. Whenever the involved mechanism belongs to spinal or supra-spinal regions (central fatigue) and if the affected structures are located distal to the neuromuscular junction (peripheral fatigue). Central fatigue is derived from alteration in the cortical cell excitability, inhibition of motor cortex output in the brain, and interruption of signal conduction in the motor neuron.

However, peripheral fatigue or neuromuscular fatigue arises from impairment in neuromuscular transmission, accumulation of metabolites such as lactate in muscle fibers, and the imbalance between intra- and extra-cellular sodium and potassium concentration incorporated with impairment in calcium release and reuptake at the sarcoplasmic reticulum. Distinguishing the fatigue into central and peripheral helps in extracting information from the recorded signal and determining the underlying cause of fatigue.

**Figure 8:**
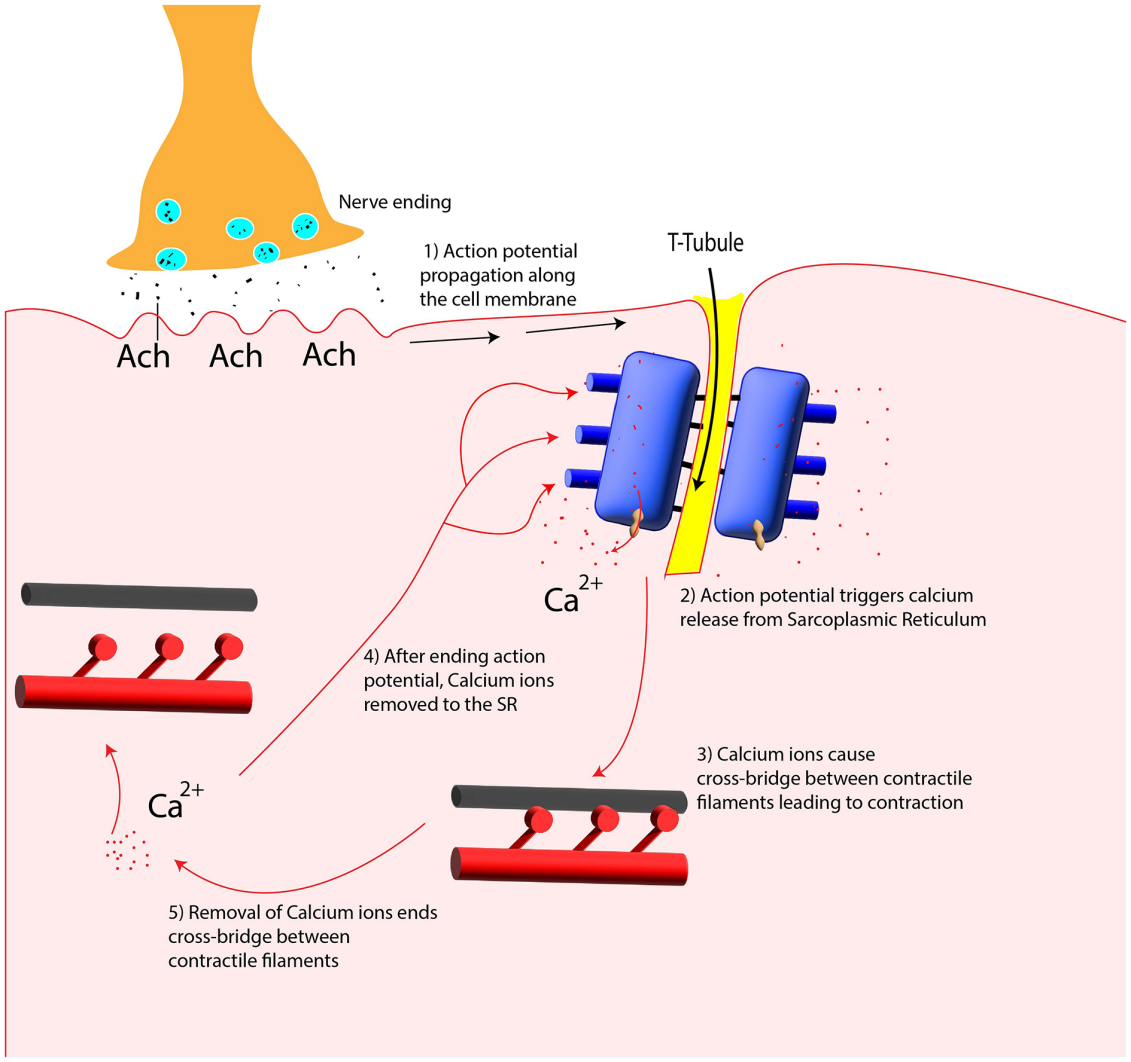
Calcium release and uptake from Sarcoplasmic Reticulum (SR) in contraction and relaxation period.

In the fatigue state, SEMG signal characteristics such as amplitude variables (Average rectified value, Root mean square) and spectral parameters (Mean and Median frequency) vary significantly. The biochemical and physiological processes in the peripheral parts which are responsible for signal changes are as follows: increased lactate concentration (deceased intramuscular PH) in sustained contraction causes a decline in muscle fiber conduction velocity (MFCV) (Cifrek et al., 2009). MFCV decline causes a decrease in mean (MNF) and median frequency (MDF). Moreover, it has been generally accepted that depolarization of the T-Tubule membrane causes the release of calcium from SR, which should be uptake during the relaxation period, as illustrated in Figure 8. The capacity of SR to uptake calcium is limited, which leads to an increase in intracellular free calcium concentration which in turn activates the Ca^2+^-dependent K^+^ channel (Sjøgaard, 1991). Activation of these channels results in an outward flux of K^+^ and a decline in membrane potential, amplitude, and conduction velocity of the intracellular action potential. Conduction velocity is not the only factor leading to the frequency decline as the observed frequency decrease can occur even without any changes in MFCV, or in proportion exceeding the MFCV changes (Cifrek et al., 2009). Although MFCV, MDF, and MNF decreased during sustained contractions, amplitude variables showed an increase (Jørgensen et al., 1988). Responsible factors that are believed to be causes of the increase in amplitude variables are synchronization between motor unit (MU) firing patterns, the recruitment of more motor units, and alternations in the firing frequency of individual motor units (Bigland-Ritchie et al., 1986).

Surface EMG is a well-established and non-invasive technique for measuring muscle fatigue. However, its amplitude and spectral parameters are affected by various factors, including electrode placement, potential current originating from the skin-electrode interface, and volume conduction effect of the surrounding tissue, which acts as a low-pass filter and can modify the signal by reducing the higher frequencies signals (Cifrek et al., 2009).

Sometti et al. (2021) recorded magnetic fields of the rectus femoris muscle in three directions (X, Y, and Z) by four OPMs placed linearly in the longitudinal axis of the muscle. After inducing fatigue, by asking participants to perform three isometric contractions for 60 s with 30 s rest in between, they observed a spectral shift toward lower frequencies, which was more noticeable in the transversal X-axis compared to the longitudinal Y-axis.

#### 4.2.5. Maximal voluntary activity alteration detected with OPM

As explained in section 2.1, with increasing muscle force, more and larger motor units are recruited, and the firing frequencies of recruited motor units also increase. Therefore, by asking subjects to perform maximal voluntary contraction, the action potentials of involved motor units are merged together and can no longer be separated by using sEMG. Therefore, due to the increase in superimposition degree of involved motor units action potential during maximal voluntary contraction (known as interference pattern/signal), the recorded interference signals are dense with similar size of amplitude (Sandbrink and Ellad, 2016).

In neurogenic disorders, muscle fibers lose their connection with the innervating motor neurons (pathology can be at any point through the entire length of the innervating motor neurons) which leading to functional impairment (Marshall and Farah, 2021). In an attempt to restore the disconnected muscle fibers activity, neighbor motor neurons sprout to the target muscle fibers, yields in an increase in the size of the remaining motor unit. Hence, in neuropathies, the interference signals will be dispersed as the number of contributed motor units decreases. Concurrently, the amplitude of the interference signal increases since the compensatory motor units innervate more muscle fibers.

In the research performed by Semeia et al. (1928), MMG signals from the rectus femoris muscle were recorded by four OPM sensors in three patients with a neuromuscular disorder. The results were inconsistent with sEMG, in which interference signals are dispersed with higher amplitude.

### 4.3. NV-based magnetometer

#### 4.3.1. Action potential detection

Barry et al. (2016) demonstrated the capability of the NV-based sensor in detecting magnetic signals in excised neurons and intact organism axons. The recorded magnetic signals waveform from both were similar, but the peak-to-peak amplitude of the excised neuron was roughly four times larger, which was in good agreement with the sensor-neuron distance. It was also proven that NV-based sensors can distinguish the direction of recorded action potential magnetic signals by modifying the stimulation point, which reveals the vector capability of NV sensors.

Muscle fiber action potential is also detected in another study (Webb et al., 2021), in which they added a compound to suppress muscle movement without inhibiting muscle action potential. They recorded muscle AP in both conditions, which can roll out the possibility that recorded magnetic signals are arising from muscle motion, not the muscle action potentials.

#### 4.3.2. Estimating conduction velocity

Conduction velocity (CV) is characterized as the propagation velocity of action potentials along the muscle fibers/axon membrane. CV is one of the fundamental physiologic properties of the muscle fiber/axon membrane, which is directly related to the muscle fiber/axon diameter. There is also a significant relation between magnetic signal amplitude and axon diameter (Kuypers et al., 1999). As such, muscle fibers/axons with smaller diameters have a lower CV and signal amplitude. At the same time, axons are tapering in radius over their length. Hence, measured magnetic signals can be modified in direction and amplitude by changing the stimulation point. In an experiment by Barry et al. (2016), the peak-to-peak amplitude of the measured magnetic field from axons is larger in posterior stimulation compared to the anterior stimulation. This asymmetry is expected to be a result of axon tapering from posterior to anterior. Therefore, NV sensors allow the detection of differences in magnetic signal amplitude due to the changes in CV and AP direction.

In another experiment by Webb et al. (2021), Muscle Fiber CV (MFCV) was roughly calculated using NV sensors, which is consistent with the MFCV range. They measured magnetic and electric biosignals from muscle simultaneously in which the NV sensors, compared to the electric probes, were placed 2 mm ±1 mm closer to the stimulation point. So magnetic signals were (1.5 ms ± 0.5 ms) ahead of the electric signals, which can give a rough estimation of the MFCV by dividing the delay between the recorded signals from two sensors over the distance between them.

### 4.4. Spintronic-based magnetometer

As mentioned before, action potential arises in the center of muscle fiber and propagates in opposite directions toward the two muscle-tendon endings. The synaptic central region has been disclosed by GMR sensor in mice soleus muscle (Barbieri et al., 2016), as the recorded magnetic signal is flat in the central region and in opposite direction at two extremities. Additionally, the recorded signal in the central region has the shortest delay and similar delays have been detected on both sides of the synaptic region.

For the first time, in an experiment conducted by Zuo et al. (2020), the TMR sensor was placed on the skin of the Abductor pollicis brevis muscle and recorded the lateral component of the magnetic field in a magnetically shielded chamber. There was a significant difference between the rest and the tense state of the muscle. During contraction, an amplitude of 200 pT magnetic field was continuously generated which is in good agreement with numerical simulation results and simultaneous EMG signals.

## 5. Discussion and future perspective

Fifty years have passed since MMG was proposed by Cohen and Gilver. There are several MMG sensors with varying performance, yet clinical approaches and studies are still limited because capturing tiny magnetic signals is still challenging. As magnetic fields decay significantly ($1/r^{3}$) with increasing distance between the sensor and signal source, MMG recordings require a high level of attentiveness to both the sensor and the signal source (skeletal muscle) geometry. Compared to other magnetic biosignals generators, like the brain in Magnetoencephalography (MEG) and the heart in Magnetocardiography (MCG), skeletal muscles are moving during contraction and relaxation, and the distance between sensor and skin is always changing, which could affect the signal amplitude and frequency (Klotz et al., 2022). Hence, a possible solution for localizing the geometry and orientation of the sensor in relation to the muscle with high precision would be implementing localization coils in several locations over the skin (a well-established method in MEG; Pfeiffer et al., 2018).

Moreover, for evaluating the effect of sensor-source distance on signal changes, muscle volume, as a key indicator, should be considered. For example, muscle volume in different skeletal muscles (Biceps Brachii muscle with large volume versus Abductor Pollicis Brevis with small muscle volume) should be considered to assess the rate of changes in recorded MMG signals in various source-skin distances.

MMG signals are the spatiotemporal summation of signals from all active motor units, and for clinical purposes, it is essential to record signals with high spatiotemporal resolution. As such, not only the ability to bring the sensor closer to the skin but determining the recording area of MMG sensors, is crucial. The recording area of each sensor depends on several sensor characteristics, like the dynamic range and the sensitivity of the sensor. These characteristics are also defined by the sensor’s readout circuit (Lenz, 1990), which should be considered in MMG recordings.

There are also technical issues related to MMG sensors that needed to be dealt with, including:

1. The need for a magnetic-shielded room or chamber for attenuating the Earth’s magnetic field and other typical magnetic noises, such as those from power lines, electronic devices, etc. Without proper shielding, these magnetic noises can interfere with the reading of MMG sensors, making it difficult to obtain accurate measurements. This can limit the versality of these sensors and the environments in which they can be used.
2. The temperature requirement for some sensors like SQUIDs (Cryocooling to −269°C) or OPM (for Rubidium ones with heating up to ∼150°C)(Tierney et al., 2019).
3. The interference between MMG sensors when they are arranged in close proximity to each other in an array configuration (Hill et al., 2020) to enhance the spatiotemporal resolution.
4. limited frequency bandwidth (the bandwidth of OPM sensors from QuSpin is 3–135 Hz), which can give rise to a loss of information that is not in the bandwidth. The required frequency bandwidth for muscle signal recordings depends on the type of muscle recording. For example, the required bandwidth for surface EMG and needle EMG is 500–600 Hz (Van Boxtel, 2001; Clancy et al., 2002) and 1 kHz (Merletti and Di Torino, 1999) respectively, as the frequency of electrical signals declines significantly while traveling through the several layers between source and skin.

The future perspectives for MMG sensors are aimed at addressing technical challenges associated with:

1. cancelation of background magnetic fields, which would allow MMG sensors to be used in a wider range of settings, without the need for a specialized magnetic-shielded room or chamber.
2. Future progress in sensor technologies that do not depend on temperature control, including spintronic-based sensors, will expand the scope of MMG application.
3. Future advancements in MMG sensor technology that do not require Helmholtz coils, such as TMR sensors, could allow for sensors to be placed in close proximity to one another without interference, enhancing the spatiotemporal resolution. This, in turn, could lead to improved accuracy and applicability of MMG sensors.
4. Improving frequency bandwidth and sensitivity is another area that can lead to greater accuracy in detecting small muscle signals. The new generation of MMG sensors, such as TMR, do not have a limitation for frequency bandwidth^12^. However, there is a trade-off between the bandwidth and the sensitivity of a magnetic sensor, which means that they cannot be optimized simultaneously, as increasing the bandwidth would reduce the sensitivity of the sensor (Bu et al., 2022).

Currently, there are only a limited number of data sets and no biologically accurate simulation models for MMG that can replicate the complex muscle structure and electrophysiological entities. As a result, there are no specific strategies for optimal MMG recordings, and the potential benefits of MMG in detecting pathologic activities remain almost unknown. However, benefiting from new technologies such as OPM and TMR with their compact size, which can operate outside the magnetically shielded room only with a portable chamber, paves the way to progress from scientific studies to clinical application. These developments can open up new opportunities to explore the potential of MMG in clinical practice.

## 6. Conclusion

We presented an overview of the neuromuscular system, underlying physiological mechanisms responsible for the electrical and magnetic signals and the state-of-the-art of several technologies used to capture magnetic signals emitted from muscles.

Regarding MMG technologies, while SQUIDs are the most sensitive sensors with the sensitivity of fT/√Hz, they suffer from several limitations, including low-temperature requirement, bulky size, and ultra-high cost. Recently, OPM came to market, which achieves Pico to femto tesla sensitivity. However, there are a few technical issues like a small dynamic range, low bandwidth, and heating requirement (for rubidium vapor) that need to be addressed in future. In addition, substantial development has been done in relation to sensors based on NV centers in diamonds. Although the sensitivity of NV-based sensors does not extend Pico tesla, they operate at room temperature and will be brought into the proximity of the target muscle due to the diamond biocompatibility. Considerable progress in nanofabrication coupled with advances in miniaturizing strategies has allowed the exciting development of spin-related magnetometers like AMR, GMR, and TMR that can be utilized as implantable or wearables due to their compact size. In terms of state-of-the-art technologies, current studies primarily focus on basic physiological studies. However, further investigations are required to fully explore the benefits of MMG in clinical settings. Overall, we conclude that MMG sensors will continue to evolve and become a promising and complementary approach for muscle activity measurement.

## Author contributions

All authors listed have made a substantial, direct, and intellectual contribution to the work and approved it for publication.

## Funding

This work was partially supported by EPSRC projects EP/X525716/1, EP/X034690/1, and EP/R004242/2. The works of NG were supported by the University of Glasgow Scholarship.

## Conflict of interest

NG, SZ, HW, KN, and HH were employed by Neuranics Ltd.

The remaining authors declare that the research was conducted in the absence of any commercial or financial relationships that could be construed as a potential conflict of interest.

## Publisher’s note

All claims expressed in this article are solely those of the authors and do not necessarily represent those of their affiliated organizations, or those of the publisher, the editors and the reviewers. Any product that may be evaluated in this article, or claim that may be made by its manufacturer, is not guaranteed or endorsed by the publisher.
